# Associations Between Nutrition Knowledge, Body Composition, and Cardiopulmonary Exercise Performance in Adolescent Football Players

**DOI:** 10.3390/sports14060231

**Published:** 2026-06-05

**Authors:** Andreea Simina Dumitrescu, Alexandru Alexandru, Sorin-Ovidiu Brîndescu

**Affiliations:** 1 Department of Kinetotherapy, Faculty of Physical Education and Sports, West University of Timișoara, Bulevardul Vasile Pârvan 4, 300223 Timisoara, Romania; andreea.dumitrescu@e-uvt.ro (A.S.D.);; 2 Neoclinic Medical Center, Calea Dorobanților 3, 307200 Timișoara, Romania; 3 Doctoral School, Victor Babes University of Medicine and Pharmacy, E. Murgu Square, No. 2, 300041 Timisoara, Romania; 4 Department of Physical Education and Sport, Faculty of Physical Education and Sports, West University of Timișoara, Bulevardul Vasile Pârvan 4, 300223 Timisoara, Romania

**Keywords:** youth football, nutrition knowledge, body composition, VO_2_max, exercise performance

## Abstract

Background: Optimizing physical performance in youth football requires a comprehensive understanding of the interplay among behavioural factors, structural body composition, and functional cardiorespiratory capacity. While sports nutrition knowledge is hypothesized to influence athletic development, its concurrent relationships with regional body compartments and objective cardiopulmonary exercise testing (CPET) metrics remain poorly characterized in adolescent athletes. Methods: A cross-sectional study approach analysed body composition via bioelectrical impedance analysis (BIA), maximal cardiorespiratory testing, and sports nutrition knowledge evaluation using the Nutrition for Sport Knowledge Questionnaire (NSKQ). Structural associations and functional predictive capacities were analysed. Results: The cohort demonstrated an average VO_2_max of 51.18 ± 16.67 mL/kg/min and a mean total nutrition knowledge score of 43.56 ± 18.06 out of 81 (53.8%). Total and domain-specific nutrition knowledge scores were not associated with body mass index (BMI), fat-free mass (FFM), or fat-free mass percentage (FFM%). Higher nutrition knowledge scores were independently associated with superior VO_2_max and anaerobic threshold (AT) metrics. Exploratory geographic analyses revealed that rural-residing participants possessed significantly higher cardiorespiratory performance values and greater baseline nutrition knowledge profiles than their urban peers. Conclusions: In adolescent male football players, sports nutrition knowledge was not associated with static body composition measures but showed exploratory positive associations with selected cardiorespiratory fitness markers. These findings should be interpreted as cross-sectional and hypothesis-generating, as some potential confounding mediators were not assessed. These findings suggest that higher sports nutrition literacy may serve as a starting point for performance-supportive behaviours and metabolic conditioning, to some degree, warranting future interventional studies.

## 1. Introduction

Football is a high-intensity intermittent sport defined by the repeated and unpredictable integration of accelerations, decelerations, changes in direction, jumps, and short maximal-effort sprints interspersed with lower-intensity locomotion [1]. Across a competitive match, outfield players cover 10–13 km, of which 800–1200 m is performed at high or very high speed, imposing substantial and fluctuating demands on aerobic, anaerobic, neuromuscular, and metabolic systems [2,3]. The aerobic system is essential for sustaining overall work rate and promoting recovery between repeated high-intensity actions, while anaerobic glycolysis and phosphocreatine resynthesis underpin the short explosive efforts—sprinting, pressing, and rapid transitions—that define decisive moments in match play [2,4]. Consequently, football performance depends not only on technical and tactical proficiency but equally on the capacity to repeatedly generate force, tolerate metabolic stress, and recover efficiently across training sessions and competition.

Within this physiological framework, body composition occupies a central role in determining football performance. Fat-free mass (FFM) directly contributes to force production, sprint acceleration, and the capacity for repeated high-intensity efforts, while excess fat mass (FM) increases the energetic cost of locomotion and attenuates relative aerobic performance [5,6,7]. In adolescent players specifically, these relationships are further complicated by marked interindividual variability in biological maturation [8,9,10]. Nutrition represents a key modifiable factor of body composition: inadequate energy availability or macronutrient imbalance can impair lean tissue accrual, accelerate muscle protein catabolism during periods of high training load, and compromise recovery—effects particularly consequential during the adolescent growth window when anabolic stimuli are especially sensitive to nutritional support [11,12,13]. Conversely, appropriate dietary practices aligned with training demands are associated with favourable body composition profiles and sustained exercise capacity [13].

Cardiopulmonary exercise testing offers an objective assessment of these physiological characteristics through variables such as maximal oxygen uptake (VO_2_max), anaerobic threshold (AT), and maximal workload. VO_2_max reflects the integrated efficiency of oxygen transport and utilization, whereas AT provides complementary information regarding the transition to greater anaerobic metabolic contribution during exercise [14,15,16,17,18]. Together, these parameters characterize both the upper and functional boundaries of aerobic performance and provide a clinically meaningful framework for assessing football readiness in young athletes. Importantly, nutrition may influence these parameters not only through its effects on body composition but also through direct effects on substrate availability, mitochondrial adaptation, and the capacity for training-induced physiological remodelling [13,19]. Yet the relationship between nutritional practices and objectively measured cardiopulmonary exercise testing (CPET) variables in adolescent footballers remains poorly characterised.

Nutritional knowledge represents a modifiable and theoretically important antecedent of dietary behaviour. The sports nutrition knowledge–behaviour relationship assumes that players with greater knowledge of energy requirements, macronutrient function, carbohydrate periodization, hydration strategies, and recovery nutrition are better equipped to make dietary choices that align intake with training demands—and therefore more likely to achieve favourable physiological adaptations [11,13,20,21]. The Nutrition for Sport Knowledge Questionnaire (NSKQ) was developed and validated to assess this domain across subcomponents including weight management, macronutrients, micronutrients, sports nutrition, and supplementation [22]. Prior work using the NSKQ and analogous instruments has consistently demonstrated that football players—including professionals—exhibit only low to moderate levels of sport-specific nutrition knowledge, with deficits in domains related to carbohydrate timing, hydration, and micronutrient function [23,24,25]. These gaps are meaningful: suboptimal knowledge in these domains may translate to chronically suboptimal dietary practice and, in turn, to less favourable body composition and attenuated physiological adaptation to training load. In adolescent athletes, living environment, habitual physical activity patterns, family structure, food availability, and opportunities for unstructured outdoor activity may all contribute to differences in both dietary behaviour and exercise adaptation independent of formal training exposure. Consequently, the effects of nutrition knowledge may not be uniform across different socio-environmental contexts.

Despite this mechanistic plausibility, empirical evidence linking nutrition knowledge to objective performance-related outcomes in youth football players is limited and methodologically inconsistent [13,26,27]. Most existing studies have relied on self-reported dietary intake—a measure subject to substantial recall and social desirability bias—and have examined body mass or body mass index (BMI) rather than the more discriminative compartmental measures of FFM and FM [12,13]. As of yet, limited data exist specifically examining the associations between NSKQ-derived nutrition knowledge and CPET-measured aerobic capacity variables in adolescent cohorts, particularly among male football players [13,26,27]. This is a meaningful omission: the link between nutrition knowledge and outcomes like VO_2_ max or AT is mediated by factors such as dietary behaviour, substrate availability, and training quality [28,29], and it remains unclear whether this pathway produces measurable effects in adolescent athletes.

Therefore, the aim of the present study was to investigate associations between nutrition knowledge, assessed using the NSKQ, and selected body composition and exercise-capacity parameters in adolescent male football players. Specifically, we examined whether total NSKQ score and domain-specific subscores were associated with body mass index, FFM, FFM%, FM, and FM%, as well as with CPET variables including VO_2_max, AT, and maximal workload. Given the multifactorial determinants of body composition and exercise performance during adolescence, we hypothesised that nutrition knowledge would demonstrate measurable associations with physiological and performance-related parameters, although the magnitude and direction of these relationships were expected to vary across body composition and aerobic capacity outcomes.

## 2. Materials and Methods

### 2.1. Study Design and Participant Selection

This cross-sectional observational study was conducted on male youth football players registered at SSU Politehnica Timișoara Sports Club during the 2024–2025 competitive season. The primary objective was to evaluate associations between nutrition knowledge, body composition, and exercise-capacity parameters in adolescent athletes. Participants were born between 2008 and 2009 and were actively engaged in structured training and competitive football at the time of assessment. Participants were additionally categorized according to living area (urban or rural residence) based on self-reported home location for exploratory subgroup analyses.

All athletes underwent a standardized assessment protocol comprising body composition analysis, CPET, and evaluation of nutrition knowledge using a validated questionnaire. To ensure data integrity, each participant contributed a single complete dataset. In cases where repeated assessments were available for the same individual, only the dataset corresponding to maximal-effort testing and complete data availability across all three domains was retained.

### 2.2. Inclusion and Exclusion Criteria

Eligibility was restricted to male football players aged 14 to 16 years who were actively engaged in structured training and official competition. Inclusion required the availability of a complete dataset encompassing body composition measurements, cardiopulmonary exercise testing results, and a fully complete nutrition knowledge questionnaire.

A complete-case analysis approach was applied; participants with missing data in any of the assessed domains were excluded from the primary analysis. Players presenting with acute musculoskeletal injuries precluding maximal-effort testing, as well as those with known chronic diseases affecting metabolic function or physical performance, were also excluded.

### 2.3. Outcome Definitions and Variables

The primary outcomes were exercise-capacity parameters obtained from CPET, including maximal oxygen uptake VO_2_max (mL/kg/min), AT (mL/kg/min), and maximal workload (W, watts). Body composition parameters constituted secondary outcomes and included FFM and FFM%, both expressed in kilograms; FM, similarly; and BMI (kg/m^2^).

VO_2_max was considered achieved when participants demonstrated volitional exhaustion together with maximal exercise criteria, including respiratory exchange ratio (RER) ≥ 1.10, attainment of age-predicted maximal heart rate, and plateau of oxygen uptake despite increasing workload.

The principal independent variable was nutrition knowledge, assessed using the NSKQ. Both the total score and individual subcategory scores were analysed, encompassing the domains of weight management, macronutrients, micronutrients, sports nutrition, and supplementation. The alcohol-related section (8 items) was excluded because the participants were minors and they also reported that they do not consume alcohol. Accordingly, the maximum achievable total NSKQ score was adjusted from 89 to 81, and all total scores were normalized to this modified scale.

Additional exploratory CPET-derived variables included VO_2_max and AT expressed relative to predicted values, oxygen pulse (VO_2_/HR), and oxygen uptake relative to estimated workload (VO_2_/W). Heart-rate variables were not included in inferential analyses because all participants fulfilled predefined maximal exertion criteria during testing.

### 2.4. Data Acquisition

#### 2.4.1. Body Composition

Body composition was assessed using bioelectrical impedance analysis (BIA) with the seca mBCA 515 body composition analyser (seca GmbH & Co. KG, Hamburg, Germany), following the standardized measurement protocols recommended by the manufacturer. Assessments were conducted under controlled conditions, with participants instructed to abstain from vigorous exercise, food intake, and fluid consumption for a minimum of 8 h prior to testing. Measurements were performed in the morning (07:00–10:00).

#### 2.4.2. Cardiopulmonary Exercise Testing

Cardiopulmonary exercise testing was performed on a motorized treadmill using a BTL CardioPoint CPET system (BTL Industries Ltd., Prague, Czech Republic), following the Bruce incremental protocol. Gas-exchange data were collected breath-by-breath and averaged over 10 s intervals. The system was calibrated prior to each test according to manufacturer guidelines. Anaerobic threshold (AT) was identified from CPET-derived gas-exchange data using the threshold value generated by the BTL CardioPoint CPET software, version 2.4 (BTL Industries Ltd., Hertfordshire, UK). The software-derived AT was based on ventilatory and gas-exchange responses during the incremental Bruce protocol. Where necessary, the AT value was reviewed for physiological plausibility using the corresponding VO_2_, VCO_2_, ventilatory equivalents, and respiratory exchange data. No separate invasive lactate-based threshold assessment was performed.

The test consisted of progressive, standardized increases in treadmill speed and inclination until volitional exhaustion. Maximal effort was confirmed using standard physiological criteria, including respiratory exchange ratio (RER) ≥ 1.10, attainment of age-predicted maximal heart rate, and plateau of oxygen uptake despite increasing workload. All participants fulfilled these criteria.

Workload values expressed in watts were automatically generated by the CPET software as estimated treadmill workload parameters derived from treadmill speed, incline, exercise stage, and participant anthropometric characteristics during the Bruce protocol. Accordingly, these variables were interpreted as exploratory exercise-capacity parameters rather than direct measures of external mechanical power output.

#### 2.4.3. Nutrition Knowledge Assessment

Nutrition knowledge was evaluated using the validated NSKQ tool, developed and validated specifically for use in athletic populations [22]. The questionnaire was undertaken in Romanian using a translated–verified version. Each correct response was assigned to one point, and both total and subcategory scores were calculated for each participant. The questionnaire was administered under standardized conditions to minimize interindividual variation in completion.

The NSKQ score was analysed across five subscales: Subscale 1 (weight management), Subscale 2 (macronutrients), Subscale 3 (micronutrients), Subscale 4 (sports nutrition), and Subscale 5 (supplementation).

### 2.5. Ethical Considerations

The study was conducted in accordance with the principles of the Declaration of Helsinki and received approval from the Ethics Committee of the CardioPrevent Foundation (Approval No. 3/28.02.2024). Prior to enrolment, written informed consent was obtained from all participants and their legal guardians. All collected data were anonymized before statistical analysis in accordance with applicable data protection regulations.

### 2.6. Statistical Analysis

Data preparation and statistical analyses were performed using Python version 3.12.7 (Python Software Foundation, Wilmington, DE, USA) with the pandas library version 2.2.3. for data cleaning and variable derivation, and IBM SPSS Statistics (version 26; IBM Corp., Armonk, NY, USA) for inferential analyses. Continuous predictor variables were standardized using z-scores prior to modelling to facilitate comparison of effect sizes across predictors, while outcome variables retained their original measurement scales. Accordingly, reported regression coefficients represent the expected change in the outcome associated with a one-standard-deviation increase in the predictor variable. Variance inflation factors (VIFs) were below 3.0 in all models, indicating no problematic multicollinearity. Regression analyses for VO_2_max and AT were not corrected for multiple comparisons and should be considered exploratory. The normality of continuous variables was assessed using the Shapiro–Wilk test.

Associations between nutrition knowledge scores, body composition parameters, and exercise-capacity variables were evaluated using Pearson or Spearman correlation coefficients, selected according to the distributional characteristics of each variable pair. Correlation effect sizes were interpreted as negligible (|r| < 0.10), small (0.10–0.29), moderate (0.30–0.49), or large (≥0.50).

Hierarchical multivariable linear regression models were constructed to examine associations between nutrition knowledge and exercise-capacity outcomes (VO_2_max, AT, and estimated maximal workload). Nutrition knowledge scores were entered in the initial model, followed by sequential adjustment for anthropometric and body composition variables. Continuous predictor variables were standardized using z-scores prior to modelling to facilitate comparison of effect sizes across predictors, while outcome variables retained their original measurement scales. Accordingly, reported regression coefficients represent the expected change in the outcome associated with a one-standard-deviation increase in the predictor variable.

Model fit was evaluated using R^2^ and adjusted R^2^ values. Multicollinearity was assessed using variance inflation factors (VIF), with values > 10 considered indicative of problematic collinearity; all observed VIF values were below 3.0. Regression analyses involving VO_2_max and AT were exploratory and were not corrected for multiple comparisons. All statistical tests were two-sided, and *p*-values < 0.05 were considered statistically significant.

### 2.7. Use of Artificial Intelligence Tools

During the preparation of this manuscript, the authors used ChatGPT (OpenAI, San Francisco, CA, USA, GPT-5.5 version) exclusively for language editing and improvement of clarity. The study design, data collection, statistical analyses, interpretation of results, and all scientific conclusions were developed by the authors, who assume full responsibility for the content of this publication.

## 3. Results

### 3.1. Study Population and Descriptive Characteristics

A total of 84 competitive youth football players were included in the study. The cohort demonstrated generally lean body composition characteristics and moderate nutrition knowledge scores. Aerobic exercise-capacity variables showed substantial interindividual variability, while most participants resided in urban areas. Full descriptive characteristics are presented in Table 1.

Nutrition knowledge performance across subscales is summarised in Table 2. Overall, participants demonstrated moderate knowledge, with a mean total score of 43.56 ± 18.06 out of 81 (53.8% of the maximum). Performance varied across domains, with the highest scores observed in Subscale 2 (59.6%), followed by Subscale 1 (55.6%) and Subscale 3 (55.3%), indicating relatively better knowledge in these areas. In contrast, lower scores were observed in Subscale 4 (45.5%) and Subscale 5 (44.5%), suggesting gaps in specific nutrition domains. Median values were broadly consistent with mean scores across all subscales.

### 3.2. Associations Between Body Composition Indicators and Nutrition Knowledge

Spearman correlation analyses were conducted to examine associations between body mass index (BMI), FFM, and nutrition knowledge scores across total and subscale measures. No statistically significant associations were identified between BMI, FFM (kg), or FFM% and any nutrition knowledge variable in the dataset (*n* = 84; all FDR-adjusted *p*-values ≥ 0.410).

Correlation coefficients were small in magnitude, ranging from ρ = 0.004 to 0.064 for BMI, from ρ = 0.143 to 0.215 for FFM (kg), and from ρ = −0.134 to 0.012 for FFM%. As shown in Table 3, body composition variables were not significantly associated with nutrition knowledge.

### 3.3. Association Between Body Composition and Physical Performance

Body composition and anthropometric variables were significantly associated with power-related performance outcomes. Height demonstrated a strong positive association with both absolute power output (ρ = 0.625, *p* < 0.001) and relative power output (ρ = 0.440, *p* < 0.001), while showing no significant association with VO_2_/W. FFM (kg) showed the strongest associations overall, with positive correlations with absolute power output (ρ = 0.702, *p* < 0.001) and relative power output (ρ = 0.545, *p* < 0.001). Body mass index (BMI) was moderately associated with absolute power output (ρ = 0.469, *p* < 0.001), relative power output (ρ = 0.405, *p* < 0.001), and VO_2_/W (ρ = 0.276, *p* ≤ 0.05).

FM (kg) was positively associated with absolute power output (ρ = 0.495, *p* < 0.001), relative power output (ρ = 0.357, *p* ≤ 0.01), and VO_2_/W (ρ = 0.340, *p* ≤ 0.01), while FM% showed similar positive associations with absolute power output (ρ = 0.404, *p* < 0.001), relative power output (ρ = 0.293, *p* ≤ 0.05), and VO_2_/W (ρ = 0.359, *p* ≤ 0.01). In contrast, FFM% was negatively associated with absolute power output (ρ = −0.404, *p* < 0.001), relative power output (ρ = −0.293, *p* ≤ 0.05), and VO_2_/W (ρ = −0.359, *p* ≤ 0.01). Table 4 summarises the associations between body composition, anthropometric variables, and performance outcomes.

### 3.4. Multivariable Modelling of Power Output

A hierarchical linear regression analysis was conducted to examine the contribution of nutrition knowledge to absolute power output, both independently and after adjustment for anthropometric and body composition variables.

In the unadjusted model (Model 1), the model explained a modest proportion of variance in power output (R^2^ = 0.153; adjusted R^2^ = 0.143; model *p* = 0.0002). In this model, nutrition knowledge was a significant positive predictor of power output (B = 35.62, *p* = 0.019).

The inclusion of height in Model 2 increased the explained variance (R^2^ = 0.214; adjusted R^2^ = 0.195; model *p* = 0.0001). Height emerged as a strong positive predictor of power output (B = 23.26, *p* < 0.001), while the effect of nutrition knowledge was attenuated and became non-significant (B = 29.74, *p* = 0.068).

In the fully adjusted model (Model 3), including FM%, model fit further improved (R^2^ = 0.270; adjusted R^2^ = 0.243; model *p* < 0.0001). Both height (B = 20.13, *p* = 0.002) and FM% (B = 21.78, *p* = 0.011) were significant positive predictors of power output. Nutrition knowledge showed a borderline association but did not reach statistical significance (B = 29.98, *p* = 0.059).

Overall, the models indicate that variability in absolute power output is driven by structural and compositional characteristics rather than nutrition knowledge alone. As summarised in Table 5, all predictor variables were z-score-standardized prior to modelling; reported B coefficients represent the expected change in the outcome (in original units) per one-standard-deviation increase in the predictor. VIF values were below 1.10 for all predictors across all models.

### 3.5. Association Between Nutrition Knowledge and Physical Performance

Hierarchical linear regression analyses were conducted to examine whether nutrition knowledge predicted aerobic and anaerobic performance outcomes, both independently and after adjustment for anthropometric and body composition variables. These analyses were exploratory and were not corrected for multiple comparisons; findings should be interpreted accordingly.

For VO_2_max, nutrition knowledge was a significant positive predictor in the unadjusted model (Model 1: R^2^ = 0.136, *p* = 0.0006; B = 6.15, *p* = 0.001). This association remained significant after adjustment for height (Model 2: R^2^ = 0.151, *p* = 0.0013; B = 6.68, *p* < 0.001; height: B = −2.10, *p* = 0.211) and after further inclusion of FM% (Model 3: R^2^ = 0.176, *p* = 0.0014; B = 6.65, *p* < 0.001; height: B = −1.71, *p* = 0.293; FM%: B = −2.69, *p* = 0.103). Neither height nor FM% reached statistical significance in any adjusted model.

For AT, nutrition knowledge was a significant positive predictor in the unadjusted model (Model 1: R^2^ = 0.089, *p* = 0.006; B = 3.81, *p* = 0.020). This association remained significant after adjustment for height (Model 2: R^2^ = 0.090, *p* = 0.022; B = 3.89, *p* = 0.024; height: B = −0.29, *p* = 0.815). In the fully adjusted model including FM%, the omnibus model did not reach conventional statistical significance (Model 3: R^2^ = 0.092, *p* = 0.052); nonetheless, nutrition knowledge remained a significant independent predictor within the model (B = 3.89, *p* = 0.025), while neither height (B = −0.37, *p* = 0.765) nor FM% (B = 0.55, *p* = 0.639) contributed significantly. Given that the overall Model 3 did not reach α = 0.05, these findings should be interpreted as exploratory.

### 3.6. Comparisons According to Living Area

Participants were classified according to living area as urban (*n* = 53) or rural (*n* = 31). Group differences were examined using Mann–Whitney U tests.

Statistically significant differences between groups were observed for several performance-related variables. Rural participants demonstrated higher values for maximal oxygen uptake (VO_2_max, mL·kg^−1^·min^−1^) (U = 503.0, *p* = 0.010, r = 0.388), AT (mL·kg^−1^·min^−1^) (U = 514.0, *p* = 0.012, r = 0.374), VO_2_max expressed as a percentage of predicted values (U = 528.0, *p* = 0.015, r = 0.357), and VO_2_ at heart rate (U = 535.5, *p* = 0.017, r = 0.348). All observed effects were of moderate magnitude.

In addition, substantial differences were observed for nutrition knowledge, with rural participants scoring significantly higher across all subscales and in total score (all *p* < 0.001, r = 0.530–0.727), corresponding to large effect sizes.

No statistically significant differences were observed between urban and rural participants for anthropometric variables (height, body mass, BMI), body composition variables (FM and FFM, expressed in both absolute and relative terms), or performance variables including power output, relative power, oxygen uptake per watt (VO_2_/W), and AT expressed as a percentage (all *p* ≥ 0.086). Across these variables, effect sizes ranged from negligible to small.

## 4. Discussion

The present study examined associations between nutrition knowledge, body composition, and cardiopulmonary exercise performance in a cohort of 84 adolescent male football players. The principal findings were threefold. First, nutrition knowledge scores—assessed using the NSKQ—were not associated with any body composition parameter, including BMI, FFM, or FFM%. Second, body composition variables, particularly FFM and height, were strongly associated with absolute power output, while no significant associations were observed between body composition and VO_2_max or AT. Third, nutrition knowledge showed exploratory positive associations with both VO_2_max and AT across regression models, an association that remained robust after adjustment for height and FM%. These findings both corroborate and complicate the existing literature and warrant careful interpretation, with consideration for the absence of data on dietary behaviour, training load, sleep, recovery, and biological maturation.

### 4.1. Nutrition Knowledge and Body Composition

The absence of a significant association between nutrition knowledge and body composition variables in the present cohort is consistent with findings reported across several athlete populations [30,31,32]. Jagim and colleagues, in a study of 67 university athletes, demonstrated that while athletes with greater nutrition knowledge tended to have lower body fat percentages, mean knowledge scores across the sample were poor—participants correctly answered 47.9% of questions on average—and the variance explained by knowledge alone was modest [30]. Similarly, Jenner and colleagues found that 46 professional Australian footballers exhibited inadequate nutrition knowledge, with an average score of 46% correct, yet formal associations with body composition were not reported [33]. The current participants scored 53.8% on average across all NSKQ domains—a marginal improvement over comparable cohorts but still within the range characterised as moderate.

Prior research across collegiate and elite sports programs demonstrates a clear deficit in sports nutrition literacy, while revealing that individual knowledge explains very little variance in actual body composition [13,30,34,35].

A theoretically important distinction exists between knowledge possession and knowledge application. The nutrition knowledge–body composition pathway is commonly modelled as indirect: knowledge shapes dietary intentions and attitudes, which in turn influence dietary behaviour, which ultimately affects body composition through energy and macronutrient balance [34,36,37,38]. This mediation may explain why cross-sectional designs consistently fail to detect direct associations between knowledge scores and composition variables. In adolescent athletes specifically, dietary behaviour is subject to strong external influences—parental food provision, team catering, peer norms, and economic access—that may override the individual-level effect of knowledge entirely, particularly in a population whose home nutrition environment is not standardized [30,32,39,40]. The current study did not assess actual dietary intake, which represents both an important limitation and a logical direction for future work seeking to decompose the knowledge–behaviour–composition pathway in youth sport populations.

### 4.2. Body Composition and Physical Performance

The significant and strong associations observed between body composition and power output—particularly for FFM (ρ = 0.702, *p* < 0.001) and height (ρ = 0.625, *p* < 0.001)—are consistent with the established literature on the determinants of maximal power production in adolescent athletes. Muscle mass is the main mechanical driver of force generation and sprint acceleration, and its association with power output in young athletes has been documented in multiple studies [28,41,42,43]. The positive association of FM and FM% with absolute power output, while counterintuitive, likely reflects the confounding effect of total body size in this age group: larger, heavier adolescents tend to carry both more fat-free and more FM, and produce higher absolute watts irrespective of composition quality [44,45]. This interpretation is supported by the finding that FFM% showed a negative association with power output, indicating that the ratio of lean-to total mass—not mass per se—is the relevant substrate.

In contrast, no significant association was detected between body composition and VO_2_max or AT. This dissociation has been described previously and is attributable in part to the relative nature of VO_2_max (expressed in mL/kg/min), which statistically corrects for body mass and thereby reduces the compositional signal [46,47]. In adolescents specifically, VO_2_max is substantially determined by cardiac output, mitochondrial density, and training-induced aerobic adaptation—factors that may be more sensitive to training history, biological maturation stage, and habitual activity patterns than to static body composition at a single measurement point [48,49,50].

### 4.3. Nutrition Knowledge and Aerobic Performance

The most novel finding of the present study was the independent, positive association of nutrition knowledge with both VO_2_max and AT, which persisted across all three regression models and was robust to adjustment for anthropometric and compositional covariates. This finding was not hypothesised a priori but is mechanistically plausible when considered through the lens of behaviour-mediated physiological adaptation.

The pathway from nutrition knowledge to aerobic performance is most logically understood as indirect. Greater knowledge of carbohydrate timing, pre-exercise fuelling, and recovery nutrition—domains captured by the NSKQ—may support more consistent substrate availability before and after training sessions, facilitating greater training quality and superior mitochondrial and cardiovascular adaptation over time [51,52,53]. This interpretation is consistent with findings by Jagim and colleagues, who reported that athletes with higher nutrition knowledge more accurately estimated their energy and carbohydrate requirements—a finding with practical implications for training load management and recovery [30].

Several non-mutually exclusive explanations may account for the observed association in the current cohort. Nutrition knowledge may additionally represent a marker for broader behavioural or environmental characteristics associated with sports participation and training adaptation: players who invest in learning about nutrition may also be more attentive to recovery, sleep, and other performance-supporting behaviours [54].

It is important to note that the regression models for VO_2_max and AT explained modest proportions of variance (R^2^ = 0.136–0.176 and R^2^ = 0.089–0.092, respectively), and that neither height nor FM% contributed independently in the adjusted models. This pattern suggests that the knowledge–performance association in this cohort may be partially confounded by unmeasured third variables—biological maturation status being the most important candidate. Biological maturation is a well-documented determinant of both aerobic capacity and the capacity to process and apply structured information in adolescent athletes [9,46,55,56,57]. More mature players may simultaneously exhibit higher VO_2_max values and greater nutrition knowledge, not because knowledge directly drives aerobic capacity, but because maturational advancement confers advantages across both domains. Future studies should include objective maturation assessment—such as skeletal age or Tanner staging—to disentangle this relationship.

### 4.4. Urban–Rural Differences in Aerobic Performance and Nutrition Knowledge

The findings regarding maximal oxygen uptake should be interpreted in the context of the specific geographic and socio-environmental characteristics of the study region. Timișoara represents a large urban centre surrounded by peri-urban and rural settlements that are functionally integrated into the metropolitan area. Children from these surrounding areas commonly attend schools within the city and are therefore exposed to broadly comparable educational systems, curricula, and socio-cultural environments. Consequently, differences observed between urban and rural groups are unlikely to reflect disparities in formal education or access to organised sports and training opportunities.

A plausible explanation for the observed differences in aerobic performance may instead relate to variations in habitual physical activity patterns. Children residing in less urbanized environments may engage more frequently in unstructured outdoor activities as part of their daily routines, including active transportation, informal play, and interaction with natural environments. Evidence from the literature suggests that younger individuals in rural settings tend to participate more in outdoor and unstructured physical activity compared to their urban counterparts [58]. Environmental context also appears to play a key role in shaping activity behaviours. Greater exposure to outdoor environments and sustained engagement in physical activity have been linked to improved cardiopulmonary fitness in children [59]. It has also been reported that children from rural or small-city settings may engage in higher levels of physical exercise compared to those from highly urbanized environments [60].

Importantly, the present findings also revealed substantially higher nutrition knowledge scores among rural participants, with large effect sizes across all subscales and total score. This result introduces an additional interpretative dimension. A potential limitation of the current study design is the possibility of self-selection bias. Participants from rural areas who were willing and able to attend a football club located in or near the city may represent a particularly motivated and engaged subgroup. Such individuals may be more committed not only to training but also to acquiring knowledge related to performance, including nutrition. Consequently, their higher nutrition knowledge may reflect greater personal investment in sport rather than differences attributable solely to living environment.

This increased knowledge may also translate into more consistent application of nutritional principles, potentially contributing to the observed differences in aerobic performance (VO_2_max and AT). In contrast, urban participants, despite having similar access to information and educational resources, may not apply nutrition knowledge to the same extent in daily practice. This discrepancy between knowledge and behaviour has been reported in previous research [30,38,61] and can partially explain the comparatively lower aerobic performance observed in the urban group.

### 4.5. Strengths and Limitations

The present study has several notable strengths. The use of CPET with the Bruce protocol provides objective, validated measurement of aerobic capacity, offering a significant methodological advance over studies relying on field-based estimates of VO_2_max. The NSKQ is a validated instrument with documented use across multiple athletic populations, supporting cross-study comparability. The complete-case analysis approach ensured data integrity, and the restriction to a single age cohort (born 2008–2009) reduces maturational heterogeneity compared to studies spanning wider age ranges.

Several limitations merit acknowledgement. The cross-sectional design precludes causal inference: associations between nutrition knowledge and aerobic performance cannot be interpreted as directional without longitudinal or interventional evidence.

The present study did not assess intermediary behavioural or contextual factors including dietary intake, recovery practices, sleep, training load, parental support, socioeconomic context, or training history. Consequently, the observed associations between nutrition knowledge and aerobic performance should be interpreted as exploratory and hypothesis-generating rather than evidence of a direct causal relationship. Biological maturation was not formally assessed, which represents an important limitation given its known influence on aerobic capacity, body composition, and developmental variability during adolescence [57].

Another important limitation should be acknowledged regarding the classification of participants by living area. Although individuals categorized as “rural” resided in officially designated rural settlements, these locations are situated in close proximity to Timișoara and are effectively part of its metropolitan area. As such, participants in the rural group may share similar socio-environmental conditions with their urban counterparts, with differences primarily relating to housing type (houses versus apartments) rather than broader structural or socioeconomic disparities typically associated with rural–urban contrasts. Consequently, the distinction between rural and urban groups in this study should be interpreted as partial rather than absolute. This consideration does not invalidate the findings but should be taken into account when interpreting the results and their generalizability.

The sample, drawn from a single club, may not generalize to other professional academies, national contexts, or female youth athletes, whose maturational and nutritional profiles differ substantially. BIA-based body composition assessment, while standardized in protocol, carries inherent measurement imprecision relative to reference methods such as DEXA, and the cross-sectional hydration status of participants—despite pre-measurement fasting instructions—cannot be fully controlled. Finally, as noted above, biological maturation was not assessed, which is a substantive omission given the known influence of maturation on both aerobic capacity and cognitive development in this age group.

## 5. Conclusions

In this cohort of adolescent male football players, nutrition knowledge was not associated with body composition, whereas anthropometric and compositional characteristics—particularly height and FFM—were strongly related to power output. By contrast, greater nutrition knowledge was independently associated with higher VO_2_max and AT, even after adjustment for body composition variables.

These findings suggest that, in youth football, nutrition knowledge may be more closely linked to aerobic fitness than to static body composition. Although causality cannot be inferred from this cross-sectional design, the observed associations support the view that nutrition knowledge may reflect, or contribute to, a broader profile of performance-supportive behaviours relevant to training adaptation.

Overall, the results identify nutrition knowledge as a potentially meaningful and modifiable correlation of exercise capacity in adolescent football players. Longitudinal studies incorporating dietary intake, behavioural measures, and biological maturation are now needed to determine whether improving nutrition knowledge can translate into measurable gains in physiological performance.

## Figures and Tables

**Table 1:** Descriptive characteristics of the study cohort (*n* = 84).

Variable	Mean ± SD	Median (IQR)	Min	Max
Height (cm)	174.98 ± 7.05	174.50 (9.00)	160.00	194.00
Body mass (kg)	63.94 ± 8.02	63.80 (13.79)	45.95	78.50
BMI (kg/m^2^)	20.82 ± 1.86	20.95 (2.62)	17.59	25.30
FFM (%)	90.80 ± 4.92	91.40 (7.40)	76.30	99.00
FM (%)	9.20 ± 4.92	8.60 (7.40)	1.00	23.70
VO_2_max (mL/kg/min)	51.18 ± 16.67	55.55 (30.26)	22.53	79.40
VO_2_max (% predicted)	91.53 ± 30.11	100.97 (56.00)	40.00	151.00
AT (mL/kg/min)	26.58 ± 12.76	25.06 (14.45)	9.65	57.30
Power output (W)	330.21 ± 91.58	315.00 (71.25)	220.00	834.00
Nutrition knowledge Score total (/81)	43.56 ± 18.06	38.00 (28.50)	14.00	81.00
Living area	Urban, *n* (%)		Rural, *n* (%)	
Distribution	53 (63.1%)		31 (36.9%)	

**Table 2:** Nutrition knowledge subscale performance (*n* = 84).

Subscale	Max Score	Mean ± SD	Median (IQR)	% of Maximum
Subscale 1	13	7.23 ± 2.19	7.00 (3.00)	55.6%
Subscale 2	30	17.88 ± 6.17	17.00 (9.25)	59.6%
Subscale 3	13	7.19 ± 3.37	7.00 (5.00)	55.3%
Subscale 4	13	5.92 ± 3.69	5.00 (6.00)	45.5%
Subscale 5	12	5.35 ± 3.55	4.00 (5.00)	44.5%
Total	81	43.56 ± 18.06	38.00 (28.50)	53.8%

**Table 3:** Spearman correlations between body composition and nutrition knowledge.

Predictor	Outcome	ρ (Range)	*p*-Value (FDR-Adjusted)
BMI	All NK variables	0.004 to 0.064	≥0.843
FFM (kg)	All NK variables	0.143 to 0.215	≥0.410
FFM (%)	All NK variables	−0.134 to 0.012	≥0.576

ρ = Spearman rank correlation coefficient; NK = nutrition knowledge; FDR = false discovery rate.

**Table 4:** Associations between body composition and performance outcomes.

Predictor	Power Output (W)	Power (%)	VO_2_/W
Height	0.625 ***	0.440 ***	NS
BMI	0.469 ***	0.405 ***	0.276 *
FFM (kg)	0.702 ***	0.545 ***	NS
FFM%	−0.404 ***	−0.293 *	−0.359 **
FM (kg)	0.495 ***	0.357 **	0.340 **
FM%	0.404 ***	0.293 *	0.359 **

NS = not significant. * *p* ≤ 0.05, ** *p* ≤ 0.01, *** *p* < 0.001.

**Table 5:** Multivariable linear regression models predicting absolute power output.

Predictor	Model 1 B (*p*)	Model 2 B (*p*)	Model 3 B (*p*)
Nutrition knowledge (total)	35.62 (0.019) *	29.74 (0.068)	29.98 (0.059)
Height (cm)	—	23.26 (0.0001) ***	20.13 (0.002) **
FM%	—	—	21.78 (0.011) *
**Statistic**			
R^2^	0.153	0.214	0.270
Adjusted R^2^	0.143	0.195	0.243
Model *p*-value	0.0002 ***	0.0001 ***	<0.0001 ***

B = regression coefficient; * *p* ≤ 0.05, ** *p* ≤ 0.01, *** *p* ≤ 0.001.

## Data Availability

The data presented in this study are not publicly available due to ethical and privacy restrictions, as the participants are minors and the identification of their affiliated sports club along with biometric data may pose a risk of re-identification. The data may be made available by the authors upon reasonable request, subject to appropriate ethical considerations and with permission from the journal editor.

## Abbreviations

The following abbreviations are used in this manuscript:

AT	Anaerobic threshold
BIA	Bioelectrical impedance analysis
BMI	Body mass index
CPET	Cardiopulmonary exercise testing
FFM	Fat-free mass
FFM%	Fat-free mass percentage
FM	Fat mass
FM%	Fat mass percentage
FDR	False discovery rate
HR	Heart rate
IQR	Interquartile range
NS	Not significant
